# Economic Challenges and Behavioral and Mental Health Risks for Overdose during the COVID-19 Pandemic among People Who Inject Drugs

**DOI:** 10.3390/ijerph19095351

**Published:** 2022-04-28

**Authors:** Leslie D. Williams, Eunhye Lee, Carl Latkin, Mary Ellen Mackesy-Amiti, Maggie Kaufmann, Elizabeth Copulsky, Charlie Kaplan, Basmattee Boodram

**Affiliations:** 1 Division of Community Health Sciences, University of Illinois at Chicago School of Public Health, Chicago, IL 60612, USA; elee247@uic.edu (E.L.); mmamiti@uic.edu (M.E.M.-A.); ecopul2@uic.edu (E.C.); bboodram@uic.edu (B.B.); 2 Johns Hopkins Bloomberg School of Public Health, Baltimore, MD 21205, USA; carl.latkin@jhu.edu; 3 Community Outreach Intervention Projects, University of Illinois at Chicago School of Public Health, Chicago, IL 60612, USA; mkaufm4@uic.edu (M.K.); kaplan21@uic.edu (C.K.)

**Keywords:** people who inject drugs, COVID-19, overdose risk, mental health, economic challenges

## Abstract

People who inject drugs (PWID) are a population that disproportionately struggles with economic and mental health challenges. However, despite numerous reports of people globally experiencing new or exacerbated economic and/or mental health challenges during the COVID-19 pandemic, the literature on the effect of the pandemic on PWID and their risk for harm (e.g., overdose) remains sparse. The present study will describe reported changes during the pandemic in risk factors for drug overdose (including changes in mental health symptoms and care access) among PWID in Chicago, and it will examine associations between such risk factor changes and the experience of economic challenges during the pandemic. Participants from an ongoing longitudinal study of young PWID from the Chicago suburbs and their injection risk network members (N = 138; mean age = 28.7 years) were interviewed about changes in their experiences, substance use behavior, and mental health since the start of the COVID-19 pandemic. Bivariate cross tabulations were computed of each “overdose risk factor” with experiences of economic challenges during the pandemic. Fisher’s Exact Tests were used to assess statistical significance. Adjusted logistic regression models were also conducted that controlled for sociodemographic characteristics, for time elapsed since the start of the pandemic, and for pre-pandemic income, homelessness, and injection frequency. Over half of our sample reported using alone more than usual during the pandemic, and over 40% reported using more than usual and/or buying drugs that were of a decreased purity or quality. Additionally, a large proportion of our sample (52.5% of those asked) reported more difficulty than usual accessing mental health care. Experiencing loss of a source of income during the pandemic was associated with using more drugs, using alone more, using a larger amount of drugs while using alone, wanting to stop using but being unable, and difficulty accessing mental health care. The preliminary associations found by the present study suggest that economic challenges or disruptions experienced during the pandemic are likely to increase risk for overdose among PWID experiencing such challenges, via changes in the above behaviors and/or conditions that are associated with risk for overdose. Intervention efforts should therefore be focused not only directly on overdose prevention, but also on assisting PWID with their economic challenges and helping them regain economic stability and access to services that may have been impeded by financial difficulty.

## 1. Introduction

The COVID-19 pandemic has precipitated numerous wide-scale social and economic changes that have affected people’s daily lives on an unprecedented global scale. People of many backgrounds have suffered and struggled in myriad ways, with impacts on microeconomics and mental health (in addition to the obvious impacts on the physical health of those infected with COVID-19) having been reported to be particularly widespread [1,2,3,4].

People who inject drugs (PWID) are a population that already disproportionately struggled with economic and mental health challenges prior to the COVID-19 pandemic. Therefore, even from the earliest days of the pandemic, there has been concern among the public health and harm reduction communities about the potential effect of the pandemic on PWID. This is in part because of the likelihood that PWID are at greater risk than non-PWID of becoming infected with COVID-19 and experiencing severe COVID-19, when infected given co-morbidities and other factors [5], but it is also because of the high likelihood that the conditions precipitated by the pandemic (including the higher likelihood of becoming infected) are likely to exacerbate already very challenging circumstances for many PWID and to thus increase risk of harm. The limited amount of data available thus far has borne out these concerns: large proportions of PWID have reported experiencing mental health challenges and concerns during the pandemic (e.g., [6]). Unfortunately, given quarantining and social distancing requirements and guidelines that were at times quite strict and limiting of people’s ability to visit public spaces, these mental health concerns are occurring in a context of additional limitations on and changes to mental health and substance use-related care and service access and availability [6,7,8]. Although evidence suggests that some care in these areas was and has been available during most of the pandemic, the changes in and limitations to services that have occurred could have created additional barriers to access for many PWID who already found access to such services challenging. Thus, given the high rates of mental health concerns among PWID and simultaneously reduced access to mental health and/or substance use care, dramatic increases in overdose and other harms have been reported during the COVID-19 pandemic. Specifically, a 42% increase between May 2019 and May 2020 has been reported in drug overdoses nationally [9], along with dramatic increases between 2019 and 2020 in both opioid overdose emergency department admissions [10] and overdose deaths that occurred at the scene (possibly due to help being less likely to be called in time or transport to a hospital being refused [11]).

Substance use behaviors are important mediators of the relationship between mental health challenges and increased risk of overdose. Such behaviors include the amount and/or frequency of use, injecting alone [12], and obtaining drugs from a new or unknown source—i.e., fentanyl is frequently found in drugs obtained from unknown or untrusted sources [13], and many PWID rely on trusted dealers to test drugs and reduce their risk of overdose [14]. Given the social, structural, and circumstantial changes and challenges created by the pandemic (e.g., social distancing and/or quarantine mandates or guidelines), it is plausible that many PWID modified their behaviors (e.g., larger volume of drugs used) in ways that may have increased their risk for overdose (e.g., injecting alone, not having access to the same drug sources). Given that such changes also occurred during the same time as the reported increases in mental health challenges experienced by PWID during the pandemic, it is also plausible that some PWID may have developed new or different mental health-related triggers (i.e., experiencing the urge to use or deciding to use based on a desire to avoid, numb, or decrease mental health symptoms) impacting their specific patterns of use. It is likewise plausible that some of the reported changes in substance use behavior may have been related to such changes in mental health, or to other different triggers or prompts for different patterns of use that resulted from the pandemic (e.g., changes in quality or purity of drugs prompting or triggering more or less frequent use due to resulting difference in speed of experiencing withdrawal).

Indeed, the limited amount of data published to date on PWID’s substance use behaviors during the pandemic provides preliminary evidence that many PWID did make such modifications. Specifically, PWID in England and Northern Ireland reported injecting more frequently, being more likely than before the pandemic to inject cocaine, and having more difficulties accessing equipment for safer injecting [15]. In Sweden and Australia, data collected from PWID about their experiences during the pandemic found that 20% of those surveyed in Sweden and 10% of those surveyed in Australia reported that the specific drug they inject had changed since the start of the pandemic [16,17]. Among the participants from the study in Sweden, 62.5% reported a decrease in the quality of heroin they were able to purchase since the start of the pandemic, and 14.7% reported insufficient access to safe injection supplies [17]. Notably, a study of PWID’s experiences during the pandemic in Baltimore found that almost 60% of those surveyed who used drugs in the last 2 weeks reported using alone, and that Black PWID were significantly more likely than non-Black PWID to report using alone [6].

However, there is a great need for additional research of this kind, and there are two gaps in this emerging body of research that should be addressed. First, there is a need for research that asks PWID from other sociocultural and geographic contexts what their behavioral experiences have been since the inception of the pandemic. In particular, in addition to a need for more data on the amount or frequency of injection and engagement in safer or riskier injection practices, there is currently a dearth of data on injection and drug use behaviors that are thought to be related to mental health challenges and/or to the social isolation created by the pandemic. For example, there is a need to understand whether the pandemic created new mental health-related triggers or worsened such triggers (e.g., perhaps due in part to social isolation created by the pandemic’s social distancing measures and/or due to increases in barriers to accessing mental health care). Second, there is a need to empirically examine the potential role of economic hardship in exacerbating mental health challenges (and thus potentially impacting drug use and injection behaviors).

Theoretical papers have discussed economic challenges and increased risk of overdose and less safe injection practices as potential or likely consequences of the COVID-19 pandemic for PWID (e.g., [5,18]). Such papers have also discussed the likely relationships between economic hardships and increases in both mental health challenges and overdoses among people who use drugs, citing several historical events in which large economic crises corresponded with rises in substance-related hospitalization and mortality [18], and an economic/political “big event” that was associated with increases in injection drug use and HIV [5]. Additionally, recent empirical research has found a relationship between unemployment and overdose mortality [19]. However, to date, empirical and modeling studies on the experiences of and additional risks facing PWID during the COVID-19 pandemic seem to have focused nearly exclusively on access to care and services (e.g., [6,15]) and/or on risk of contracting COVID-19 (e.g., [6,15,20]) or HIV (e.g., [21,22]). To our knowledge, no empirical studies have yet examined whether there is a direct relationship between pandemic-based economic challenges and reported changes in risk for substance use or overdose among PWID.

To address the gaps in the literature described above, the present study will address the following research questions among a sample of young PWID—a population at increased risk for drug overdose deaths and mental illness [23,24]—residing in Chicago, Illinois and the surrounding suburbs, and their injection network members of any age (mean age of full sample = 28.7 years):

(1) What changes were reported in substance use behaviors that are posited to increase risk for overdose among young PWID and their injection network members during the COVID-19 pandemic?

(2) Is there a relationship between individuals’ pandemic-related socioeconomic changes (e.g., loss of job or income) and reported change in behaviors that confer risk for overdose?

We hypothesized that a substantial proportion of our participants would report a number of changes in their substance use behavior since the start of the pandemic that would confer greater risk of overdose, such as using drugs more often, using alone more often, and using larger amounts than before the pandemic. We also hypothesized that participants who reported socioeconomic challenges such as loss of job or income would be more likely to report an increase in behaviors that confer greater risk for overdose.

## 2. Materials and Methods

### 2.1. Sample

We used data from a longitudinal network-based study of young (aged 18–30) PWID and their injection network members (of all ages). To be eligible, ego participants (i.e., initial participants who were asked to recruit their network members) had to be (i) 18–30 years old, (ii) current injectors (i.e., injected ≥ 1 in past 30 days), (iii) willing to recruit their injection network alters who were ≥18 years old at baseline and 24 months, (iv) willing to be tested for HIV and HCV, and (vii) residing in the Chicago Metropolitan Statistical Area in the past 12 months. The injection network members (i.e., alters) of the egos were eligible if they were (i) ≥18 years old, (ii) current PWID, and (iii) had injected drugs with the ego in the past 6 months. Current injector status was verified by experienced study staff inspecting injection stigmata and, if questionable, using a standardized procedure from earlier studies to evaluate participant knowledge of the injection process. Age was verified with a driver’s license or a state ID card. Project staff offered to assist those without identification in obtaining it.

For the present study, a subsample of participants was selected who were available to complete surveys, on or after 29 April 2020, asking questions about their experiences after the start of the COVID-19 pandemic. Of our 280 baseline participants (169 egos and 111 alters), we were able to collect such data from 138 participants. One hundred of these (72.5%) had already completed baseline data for the project before the start of the pandemic, and were asked to complete the pandemic-specific items in follow-up surveys. The other 38 participants completing these items completed baseline data collection after the start of the pandemic.

### 2.2. Recruitment, Enrollment, and Compensation

Recruitment and baseline data collection for the proposed study was conducted at two field sites of a community outreach center located in Chicago that has been providing services (e.g., syringe service programs; HIV and HCV counseling, testing, and case management) and conducting research on people who use illicit substances for over 30 years. These field sites attract both urban and suburban PWID, and are located in areas that have rates above the city’s average for HIV/AIDS, sexually transmitted infections, viral hepatitis, and arrests for drug-related offenses. We recruited most egos from the syringe services program (SSP) at these field sites. To enhance the representativeness of the suburban PWID population by targeting non-SSP suburban PWID, a sizeable proportion of egos were also recruited via other methods: direct recruitment in drug market areas and at community fairs using an outreach van; fliers posted at community-based organizations serving PWID; social media and other online ads.

To reduce bias and ensure a diverse sample, we used three approaches to recruit ego participants. First, we approached SSP participants daily to screen for eligibility and offer enrollment. Second, SSP-recruited participants were screened to ascertain if they obtained syringes at the SSP for other people who reside in the suburbs. We offered those who said yes a coupon to refer to the study an age-eligible peer who did not use the SSP or purchase/use drugs in Chicago. To encourage peer-recruited PWID to participate, we used a mobile outreach van staffed with an interviewer/phlebotomist, to conduct data collection off-site near the recruit’s residence or other mutually agreed upon locations. Third, screening and enrollment of non-SSP PWID from drug market areas were conducted by indigenous field staff with extensive experience working in these areas and recruiting for similar studies.

At baseline, we asked participants to complete a survey about the members of their injection networks, their injection and other risk behaviors, HIV and HCV testing history, perceptions of stigma, mental health symptoms they might be experiencing, and a set of sociodemographic characteristics. At follow-ups starting on 29 April 2020, we asked participants a set of additional questions about changes in economic circumstances, social distancing and other COVID-19 preventive measures, and changes in substance use, behaviors and/or mental health concerns since the start of the pandemic. Because the time needed to complete surveys that ask about social networks varies considerably depending on network size, we compensated participants hourly (USD 20 per hour; average time of about 2 h). Most participants completed the survey within a 2.5 h session that included a break (average USD 50).

At their baseline visit, we asked each ego participant to recruit up to five alters (i.e., people they injected drugs with at least once in the prior six months) using recruitment coupons that provided information about the study and were linked to the recruiting ego via alphanumeric code. Coupons could only be redeemed by alters named by an ego participant during their survey. Data collection from alters was required to occur within 6 months of the ego’s baseline visit. We recruited a total of 111 alter participants. In addition to hourly compensation for interviews, egos were reimbursed USD 20 for each alter enrolled, to compensate them for the time and effort expended to recruit their network members. For completion of the additional follow-up survey about COVID-19 and participants’ experiences during the pandemic, we also compensated participants an additional USD 15.

All participants (alters and egos) completed a process of informed consent. All participants were also tested for HCV and HIV. Blood samples were collected from participants for this purpose, and appropriate counseling was provided to accompany these tests. All COIP services (e.g., SSP; HCV and HIV testing, counseling, and case management; linkage to medical care) were made freely available to all PWID screened, regardless of study enrollment. All study procedures were approved by an Institutional Review Board at the University of Illinois at Chicago.

### 2.3. Measures

Injection Behavior Changes and Indicators of Potential Risk for Overdose.

At follow-up interviews occurring between 29 April 2020 and 28 June 2021, participants were asked a series of questions about whether and how their specific injection behaviors (e.g., amount and frequency of injection; injecting alone or with others; desire or urges for more frequent injection; triggers prompting injection), mental health concerns, access to care and services, and sources of acquiring drugs had changed between the time before the “start” of the pandemic in the United States (i.e., roughly March 2020) and the time of the interview. Participants were prompted to think specifically about changes that they thought were at least in part attributable to the pandemic or to pandemic-related events or circumstances. Some of the items were asked with dichotomous response options (i.e., “Did you experience new or worsening mental health concerns or problems since the start of the pandemic?” with response options of “Yes” or “No”). Other items asked participants to characterize changes as either increases or decreases (e.g., “How would you characterize your frequency of injecting drugs since the start of the pandemic, compared to before the pandemic started?” with response options of “Did not change;” “Decreased a little”, “Decreased somewhat”, “Decreased a lot”, “Increased a little”, “Increased somewhat”, or “Increased a lot”). See Supplementary Materials for a list of all items and response options used for the dependent variables. Due to sparseness of participants in each cell (i.e., selecting each response option), and for consistency of analyses across items, we dichotomized all items for the present study, such that a “Yes” is indicative of a reported change that would be theoretically likely to increase risk for overdose (e.g., a reported decrease in purity of drugs purchased, a reported decrease in access to mental health care, a reported increase in mental health challenges, or a reported increase in injecting drugs alone), and such that a “No” is indicative either of no reported change or of a reported change that would be theoretically likely to decrease risk for overdose.

Indicators of Economic Instability During the COVID-19 Pandemic.

As part of the same interview section in which participants reported on changes they experienced during the pandemic in their injection behavior, mental health, and other risks for overdose, we also asked participants to respond to three dichotomous items that asked them whether or not they experienced major economic changes/challenges since the start of the pandemic. Specifically, we asked them whether they (1) experienced a change in their primary source of income, (2) experienced a loss of at least one source of income, and (3) experienced a change in their housing situation since the pandemic started (i.e., since March 2020).

### 2.4. Analyses

Using SPSS (v. 21; IBM SPSS Statistics, Armonk, NY, USA), frequencies and descriptive analyses were conducted for each of the economic challenge and “risk for overdose” variables of interest described above. Given the exploratory nature of our analyses, cross tabulations were computed to examine bivariate associations between each of the economic change variables and each of the “risk for overdose” variables. Bivariate associations were identified using the *p*-values from Fisher’s Exact Tests, given the potential bias that could be introduced by using chi-square tests among such a small sample, and with cross tabulations containing small cell sizes. We therefore report *p*-values from Fisher’s Exact Tests. No adjustments were made for multiple comparisons since such adjustments may lead to increases in Type II error, given that we expect the phenomena in question to be associated based on theoretically supported relationships, and not based only on chance [25].

We also conducted adjusted analyses to account for important potential covariates and to better estimate the potential relationships of interest. Specifically, we conducted a series of adjusted binary logistic regression models, each of which included (a) one of our “risk for overdose” variables of interest as an outcome, each of which included (b) one of our economic challenges variables as the primary predictor of interest, and (c) the following covariates: age, gender, race/ethnicity, Cook county residence, baseline (pre-pandemic) homelessness, baseline (pre-pandemic) income, baseline (pre-pandemic) frequency of injection, and amount of time passed since the beginning of the pandemic (measured in 3-month increments based on the variability among our sample). Since some of our sample enrolled in the study after the pandemic began, we only had baseline (pre-pandemic) covariate measures for 92 of our 138 participants, resulting in an N of 92 for our adjusted analyses. Since this relatively small N limits our statistical power for adjusted models with eight covariates, we discuss “marginally significant” (*p* < 0.10) associations in addition to significant (*p* < 0.05) associations among our results.

## 3. Results

### 3.1. Descriptive Analyses

Table 1 provides a summary of the sociodemographic characteristics of the sample, and their self-reported history of COVID-19 positivity. Descriptive analysis of the set of indicators of potential risks for overdose revealed that large proportions of our sample perceived increases in risk behaviors, barriers to care, and mental health challenges or symptoms during the pandemic (i.e., since the start of the pandemic). Table 2 presents these findings. Specifically, over half (50.4%) of respondents reported using more by themselves more frequently than usual during the pandemic (i.e., since the start of the pandemic), and said that it had been more difficult to access mental health services (52.5%). Nearly half of respondents reported wanting to use more since the start of the pandemic (48.1%), actually using more than usual since the start of the pandemic (42.3%), having different triggers for using than normal (40.6%), and buying drugs since the start of the pandemic that were of a decreased purity (40.3%) and of a decreased quality (40.6%), relative to what they usually buy. A quarter or more of participants reported experiencing new or worse mental health symptoms or problems since the start of the pandemic (24.6%), injecting a greater number of times per day since on average since the start of the pandemic (28.3%), believing there was more fentanyl or a greater probability of there being fentanyl in the drugs they had purchased since the start of the pandemic (31.1%), using a larger amount of drugs when using by themselves since the start of the pandemic (28.1%), and having a larger amount of triggers for using than they had before the start of the pandemic (27.5%). More than one fifth of our participants reported an increase, since the start of the pandemic, in trying to stop using, but being unable (22.0%).

Likewise, large proportions of the present sample reported experiencing economic hardships during the pandemic. Among our sample of young PWID and their network members, 60.1% reported experiencing a change in their primary source of income due to the pandemic, 50.0% reported experiencing a loss of at least one source of income due to the pandemic, and 14.5% reported experiencing a change in their housing situation due to the pandemic.

### 3.2. Bivariate Analyses

Cross tabulations were computed between each of the economic change variables and each of the “risk for overdose” variables, separately. These results are presented in Table 2. Associations were identified using the *p*-values from Fisher’s Exact Tests, given the potential bias that could be introduced by using chi-square tests among such a small sample, and with cross tabulations containing small cell sizes.

#### 3.2.1. Change in Primary Source of Income

Among PWID, change in primary source of income during the pandemic was significantly associated with both using more than usual (*p* = 0.032), and with participants’ using more by themselves than usual (*p* = 0.013). Change in primary income during the pandemic was also marginally significantly associated with (i.e., had a trend-level association with) wanting to use more (*p* = 0.074), with being worried about overdosing (*p* = 0.089), with reporting an increase in worry about overdose since the start of the pandemic (*p* = 0.066), and with trying to stop but being unable (*p* = 0.085).

#### 3.2.2. Loss of Source of Income

Loss of a source of income since the pandemic started was significantly associated with using more than usual (*p* = 0.011), with having more difficulty accessing mental health services (*p* = 0.025), with using a larger amount of drugs while alone (*p* = 0.048), and with trying to stop but being unable to (*p* = 0.036). Loss of a source of income since the pandemic started was also marginally significantly associated with (i.e., had a trend-level association with) wanting to use more (*p* = 0.070) and with having different triggers than normal (*p* = 0.084).

#### 3.2.3. Change in Housing Situation

Change in housing situation since the start of the pandemic was also marginally significantly associated with (i.e., had a trend-level association with) reporting worrying about overdosing (*p* = 0.057) and with reporting greater than usual difficulty accessing mental health services (*p* = 0.062).

### 3.3. Adjusted Logistic Regression Analyses

Results from all adjusted binary logistic regression models are presented in Table 3.

#### 3.3.1. Change in Primary Source of Income

Similar to the bivariate findings, adjusted logistic regression models controlling for sociodemographic characteristics, time elapsed since the start of the pandemic, and baseline (pre-pandemic) income, homelessness, and injection frequency found that reported changes in primary income were significantly associated with participants’ using more by themselves than usual (OR = 2.82; *p* = 0.032), and were marginally significantly associated with using more than usual (OR = 2.31; *p* = 0.093) and with being worried about overdosing (OR = 5.24; *p* = 0.076). Additionally, a marginally significant association was found using adjusted models, between reported changes in primary income and reported increases in the number of times injected per day (OR = 2.82; *p* = 0.086).

#### 3.3.2. Loss of Source of Income

Similar to the bivariate findings, adjusted logistic regression models found that loss of a source of income since the pandemic started was significantly associated with using more than usual (OR = 2.63; *p* = 0.043), and with using a larger amount of drugs (OR = 3.89; *p* = 0.017), and was marginally significantly associated with trying to stop but being unable to (OR = 2.67; *p* = 0.099).

We were unable to test whether the bivariate association found between loss of source of income and having more difficulty accessing mental health services would have also been found using an adjusted model, because only participants who reported having prior access to mental health services or needing mental health services were asked this question. The resulting N of 30 for this variable among participants who also had pre-pandemic baseline data on covariates of interest was insufficient to conduct the desired adjusted model.

#### 3.3.3. Change in Housing Situation

Similarly to the bivariate findings, change in housing situation since the start of the pandemic was significantly associated with reporting worrying about overdosing (OR = 8.76; *p* = 0.032). Additionally, marginally significant associations were found using adjusted models, between reported changes in housing situation and reported decreases in both purity of drugs purchased (OR = 0.22; *p* = 0.078) and quality of drugs purchased (OR = 0.15; *p* = 0.050), but in an unexpected direction: those who reported changes in housing situation were marginally significantly less likely to report decreases in drug purity and quality.

We were unable to test whether the bivariate association found between change in housing situation and having more difficulty accessing mental health services would have also been found using an adjusted model, due to the small N for this variable, described above.

## 4. Discussion

### 4.1. Reports of Increased Overdose Risks among PWID during the First 15 Months of the Pandemic

Consistent with the limited amount of extant literature examining changes in PWID’s risk behavior during the pandemic [15,16,17], the present study found that large proportions of young PWID and their network members in Chicago and the surrounding suburbs had increased behaviors associated with higher risk for overdose. Specifically, over half of our sample reported using alone more than usual, and over 40% reported using more than usual and/or buying drugs that were of a decreased purity or quality. Additionally, a large proportion of our sample reported more difficulty than usual accessing mental health care, which could also increase risk for overdose. These descriptive findings alone (in combination with the extant findings that overdoses have in fact increased dramatically during the pandemic [9,10,11]) suggest that harm reduction efforts among PWID should be increased as the pandemic continues.

This study was the first, to our knowledge, to test for a relationship between economic challenges experienced during the pandemic and factors associated with increased risk for overdose among PWID. Although preliminary and exploratory, the findings suggest that experiencing loss of a source of income during the pandemic was in fact associated with a set of specific factors that could increase risk for overdose, including using more, using alone more, using a larger amount of drugs while using alone, wanting to stop using but being unable, and difficulty accessing mental health care. This suggests that economic challenges or disruptions experienced during the pandemic are likely to increase risk for overdose among PWID experiencing such challenges, via these mediating risk behaviors and/or conditions.

PWID were at high risk for experiencing mental health challenges and overdose even before the pandemic. The present findings and extant literature suggest that this risk has increased during the pandemic, on both counts. Given the association found between economic challenges during the pandemic and increases in these behaviors and conditions that are associated with increased risk for overdose, intervention efforts should be focused not only on direct harm reduction efforts, but also on assisting PWID with their economic challenges and helping them regain economic stability (e.g., employment, for cases in which employment was lost during the pandemic) and access to services (e.g., mental health care) that may have been impeded by financial difficulty.

### 4.2. Limitations

Given the challenges of collecting data during the pandemic, our interviews were quite spread out in time, resulting in a large degree of variation between participants in the amount of time that had passed between the start of the pandemic and the participants’ interviews. Therefore, while some participants in our sample are reflecting on experiences occurring early on during the pandemic (as early as late April 2020), others are likely reflecting on experiences occurring much more recently (e.g., as late as June 2021, which is about fifteen months after the start of pandemic-related quarantine in the U.S.). Though we did control for this variation in timing and “stage of the pandemic” in our adjusted models, future research should aim to more systematically examine variation in experiences by specific periods of time. Additionally, our adjusted models were limited in their statistical power to detect significant associations, due to our relatively small sample size and the inclusion of eight covariates. Additionally, we were unable to control for a history of having been infected with (or having been ill with) COVID-19, since we added items assessing this to our survey later in our study, after we had already surveyed almost two thirds of our participants (see Table 1). We did assess this among 48 (34.8%) of our participants, among whom only 2 indicated that they had ever had COVID-19. This was insufficient variability for inclusion in our adjusted models, and is therefore a limitation of these models.

Despite our interest in changes in circumstances and behavior experienced during the COVID-19 pandemic (i.e., as compared to the experiences and behaviors of our participants before the start of the pandemic), our data are cross-sectional. Unfortunately, we did not ask participants about many of the phenomena we examine here before the start of the pandemic, and as such have no baseline measures with which to conduct a longitudinal analysis. We have simply asked participants to reflect on and recall their own experiences, circumstances, and behaviors both before and after the start of the pandemic, and to draw their own comparisons and make their own conclusions about how things have changed for them since the pandemic began. This method of data collection has obvious limitations and potential sources of bias. Recall bias is likely to be great, and even among participants with excellent memories, comparisons drawn are likely to be flawed and biased to emphasize certain features of their experiences, and are also likely to be influenced by social factors such as norms and zeitgeist (i.e., the pervasive idea that the pandemic has made things hard for people, generally). Social desirability bias may even come into play in participants’ responses, as they may seek to use the pandemic to justify current behaviors that they fear may be judged by data collectors (and/or of which they may be self-critical). Our hope is that there are some ongoing longitudinal projects which have happened to capture some of these same constructs among PWID before the start of the pandemic, and which have also (and/or will again) capture them since the start of the pandemic, such that direct longitudinal comparisons can be drawn between equivalent measures both before and after the start of the pandemic, in order to draw more confident conclusions about the pandemic’s effects on the experiences and behaviors of PWID.

Finally, we acknowledge that the external validity of our findings is limited by our sampling strategy. The associations we found are not generalizable to PWID in other geographic areas, to PWID of different racial/ethnic, cultural, or linguistic backgrounds, or to PWID of different ages (i.e., older PWID).

## 5. Conclusions

The data presented here represent an important preliminary investigation into the economic challenges experienced by PWID during the COVID-19 pandemic, and the relationship of such challenges to behavioral, social, and service access factors that can be considered indicative of increased risk for overdose. The present findings suggest that the experience of challenging economic changes (e.g., loss of income or change in housing status) was associated with a greater likelihood of engaging in riskier substance use behavior and of experiencing more or worse mental health challenges and/or triggers for substance use, and with a decreased ability to access mental health care, among PWID in the greater Chicago area. The simultaneous experience of these phenomena (economic hardship, mental health challenges, lack of access to services, and “riskier” substance use) in individuals during the COVID-19 pandemic could certainly amount to increased risk of overdose for many young PWID experiencing this “perfect storm” of both new challenges and exacerbation of existing challenges. Though some features of life (e.g., accessing care/services) may have become easier now than they were early in the pandemic, outreach interventions are needed that link PWID to harm reduction services, mental health care, and sources of economic support, to holistically address the multiple domains of well-being that have been impacted by the pandemic, among a large portion of this population.

## Figures and Tables

**Table 1:** Sociodemographic characteristics of young people who inject drugs (PWID) and their network members.

	N	%
Gender		
Male	96	69.6%
Female	37	26.8%
Transgender	1	0.7%
Missing Data	4	2.9%
Age		
18–24	14	10.1%
25–30	91	65.9%
31+	30	21.7%
Missing Data	3	2.2%
Race/ethnicity		
Non-Hispanic Black	7	5.1%
Hispanic	41	29.7%
Non-Hispanic White	79	57.2%
Non-Hispanic Other or Mixed Race	7	5.1%
Missing Data	4	2.9%
City of Residence		
Chicago	53	38.4%
Other, suburban	85	61.6%
Missing Data	0	0.0%
History of COVID-19 Positivity		
Yes	2	4.2%
No	46	95.8%
Not asked, because we introduced this question into the survey after these participants had already completed it	90	
Timing of Interview		
April 2020–June 2020	29	21.0%
July 2020–September 2020	35	25.4%
October 2020–December 2020	30	21.7%
January 2021–March 2021	19	13.8%
April 2021–June 2021	25	18.1%

**Table 2:** Results of Fisher’s Exact Tests of Associations between Economic Challenges Experienced during the COVID-19 Pandemic and Reported Changes in Risk Factors for Overdose (N = 138).

	N (Valid %) of Participants Experiencing Risk	N Missing	Cross Tabulation by Change in Primary Source of Income: (*p*-Value from Fisher’s Exact Test)	Cross Tabulation by Loss of Source of Income: (*p*-Value from Fisher’s Exact Test)	Cross Tabulation by Change in Housing Situation: (*p*-Value from Fisher’s Exact Test)
Reported Wanting to Use More than before Pandemic *	64 (48.1%)	5	(*p* = 0.074)	(*p* = 0.070)	(*p* = 0.294)
Reported Actually Using More than Usual *	55 (42.3%)	8	(*p* = 0.032)	(*p* = 0.011)	(*p* = 0.405)
Reported Using More by Themselves than Usual *	65 (50.4%)	9	(*p* = 0.013)	(*p* = 0.167)	(*p* = 0.169)
Reported Worrying about Overdosing During the Pandemic *	21 (16.2%)	8	(*p* = 0.089)	(*p* = 0.500)	(*p* = 0.057)
Reported Having Different Triggers for Using than Usual	54 (40.6%)	5	(*p* = 0.122)	(*p* = 0.084)	(*p* =0.246)
Reported Experiencing New or Worsening Mental Health Problems or Symptoms	34 (24.6%)	1	(*p* = 0.242)	(*p* = 0.547)	(*p* = 0.775)
Reported Having More Difficulty Accessing Mental Health Services	21 (52.5%)	98	(*p* = 0.107)	(*p* = 0.025)	(*p* = 0.062)
Reported Increasing the Number of Times Injected Per Day *	36 (28.3%)	11	(*p* = 0.329)	(*p* = 0.336)	(*p* = 0.464)
Reported a Decrease in Purity of Drugs Purchased *	52 (40.3%)	9	(*p* = 0.552)	(*p* = 0.205)	(*p* = 0.136)
Reported a Decrease in Quality of Drugs Purchased *	52 (40.6%)	10	(*p* = 0.529)	(*p* = 0.429)	(*p* = 0.341)
Reported More Fentanyl in Drugs Purchased *	38 (31.1%)	16	(*p* = 0.325)	(*p* = 0.529)	(*p* = 0.121)
Reported Amount of Drugs Used Alone Has Increased Since Start of Pandemic *	36 (28.1%)	10	(*p* = 0.413)	(*p* = 0.048)	(*p* = 0.546)
Reported that Amount of Worrying about Overdosing Has Increased *	22 (17.2%)	10	(*p* = 0.066)	(*p* = 0.267)	(*p* = 0.581)
Reported Having More Triggers than Normal *	36 (27.5%)	7	(*p* = 0.238)	(*p* = 0.487)	(*p* = 0.488)
Reported Trying to Stop but Being Unable *	28 (22.0%)	11	(*p* = 0.085)	(*p* = 0.036)	(*p* = 0.411)

* Original item was collapsed into a dichotomous variable from an original response scale that had more than two options. See Section 2.3 for basic description of response scales, and see Supplementary Materials for detailed item and response scale descriptions.

**Table 3:** Results of Adjusted Logistic Regression Models ** Assessing Associations between Economic Challenges Experienced during the COVID-19 Pandemic and Reported Changes in Risk Factors for Overdose (N = 92).

Outcome Variable	N (Valid %) of Participants Experiencing Risk	N Missing	Regression Predictor: Change in Primary Source of Income: Odds Ratio (*p*-Value)	Regression Predictor: Loss of Source of Income: Odds Ratio (*p*-Value)	Regression Predictor: Change in Housing Situation: Odds Ratio (*p*-Value)
Reported Wanting to Use More than before Pandemic *	42 (45.7%)	0	1.41 (*p* = 0.343)	1.70 (*p* = 0.240)	0.52 (*p* = 0.337)
Reported Actually Using More than Usual *	37 (41.6%)	3	2.31 (*p* = 0.093)	2.63 (*p* = 0.043)	1.90 (*p* = 0.365)
Reported Using More by Themselves than Usual *	42 (48.3%)	5	2.82 (*p* = 0.032)	1.60 (*p* = 0.299)	1.87 (*p* = 0.365)
Reported Worrying about Overdosing During the Pandemic *	13 (14.8%)	4	5.24 (*p* = 0.076)	1.79 (*p* = 0.442)	8.76 (*p* = 0.032)
Reported Having Different Triggers for Using than Usual	35 (38.5%)	1	1.31 (*p* = 0.579)	1.88 (*p* = 0.182)	1.47 (*p* = 0.561)
Reported Experiencing New or Worsening Mental Health Problems or Symptoms	23 (25.0%)	0	1.59 (*p* = 0.379)	1.48 (*p* = 0.442)	2.01 (*p* = 0.313)
Reported Having More Difficulty Accessing Mental Health Services	17 (56.7%)	62	Unable to test due to small N	Unable to test due to small N	Unable to test due to small N
Reported Increasing the Number of Times Injected Per Day *	24 (28.2%)	7	2.82 (*p* = 0.086)	2.19 (*p* = 0.162)	1.87 (*p* = 0.408)
Reported a Decrease in Purity of Drugs Purchased *	38 (43.7%)	5	1.42 (*p* = 0.491)	1.90 (*p* = 0.203)	0.22 (*p* = 0.078)
Reported a Decrease in Quality of Drugs Purchased *	38 (44.2%)	6	1.34 (*p* = 0.584)	1.10 (*p* = 0.856)	0.15 (*p* = 0.050)
Reported More Fentanyl in Drugs Purchased *	27 (32.1%)	8	1.23 (*p* = 0.696)	0.73 (*p* = 0.538)	0.35 (*p* = 0.220)
Reported Amount of Drugs Used Alone Has Increased Since Start of Pandemic *	25 (29.1%)	6	2.23 (*p* = 0.150)	3.89 (*p* = 0.017)	1.24 (*p* = 0.773)
Reported that Amount of Worrying about Overdosing Has Increased *	13 (15.1%)	6	1.82 (*p* = 0.467)	1.47 (*p* = 0.615)	1.53 (*p* = 0.673)
Reported Having More Triggers than Normal *	26 (29.2%)	3	0.47 (*p* = 0.149)	0.69 (*p* = 0.473)	0.84 (*p* = 0.811)
Reported Trying to Stop but Being Unable *	20 (23.5%)	7	2.84 (*p* = 0.107)	2.67 (*p* = 0.099)	1.90 (*p* = 0.396)

* Original item was collapsed into a dichotomous variable from an original response scale that had more than two options. See Section 2.3 for basic description of response scales, and see Supplementary Materials for detailed item and response scale descriptions. ** All analyses adjusted for age, gender, race/ethnicity, Cook county residence, time elapsed since start of pandemic, baseline (pre-pandemic) income, baseline (pre-pandemic) homelessness, and baseline (pre-pandemic) injection frequency.

## Supplementary Materials

The following supporting information can be downloaded at: https://www.mdpi.com/article/10.3390/ijerph19095351/s1, Online Supplement for “Economic Challenges and Behavioral and Mental Health Risks for Overdose during the COVID-19 Pandemic among People Who Inject Drugs”.
