# Supplementary material for: Economic Challenges and Behavioral and Mental Health Risks for Overdose during the COVID-19 Pandemic among People Who Inject Drugs
Source: Int J Environ Res Public Health. 2022 Apr 28;19(9):5351. doi: 10.3390/ijerph19095351 (PMC9101403; doi:10.3390/ijerph19095351)
Supplement: Supplementary file 1 [file ijerph-19-05351-s001.zip › ijerph-1659418-supplementary.pdf]

Online Supplement for "Economic Challenges and Behavioral and Mental Health Risks for Overdose during the COVID-19 Pandemic among People Who Inject Drugs"

Items Measuring Independent Variables

Have you experienced a change in your primary source of income because of COVID-19 (since March 2020)?

Yes | No | N/A | Don't know

Have you lost any sources of income because COVID-19 (since March 2020)?

Yes | No | N/A | Don't know

Has your housing situation changed because of COVID-19 (since March 2020)?

Yes | No | N/A | Don't know

Items Measuring Dependent Variables

Have you experienced any of the following because of COVID-19 (since March 2020):

-Wanted to use more
No | Yes, a little | Yes, somewhat | Yes, a lot | N/A | Don't know

-Used more than usual

No | Yes, a little | Yes, somewhat | Yes, a lot | N/A | Don't know

-Used more by yourself than usual

No | Yes, a little | Yes, somewhat | Yes, a lot | N/A | Don't know

-Worried about overdosing

No | Yes, a little | Yes, somewhat | Yes, a lot | N/A | Don't know

-Had different triggers (ex. things that can make you want to use) for using than normal

No | Yes, a little | Yes, somewhat | Yes, a lot | N/A | Don't know

Since the COVID-19 pandemic (since March 2020), have you experienced any new or worsening emotional or mental health problem?

Yes | No | N/A | Don't know

Please rate how difficult it was for you to access each of the following because of COVID-19 over the past month. Because of COVID-19, my access to drug or alcohol treatment over the past month has been...

Not at all difficult | Somewhat difficult | Difficult | Very difficult | N/A

How has the number of times you inject per day changed during COVID-19 (since March 2020)?

Increased a lot | Increased somewhat | Increased a little | Stayed the same | Decreased a little | Decreased somewhat | Decreased a lot | N/A

Because of COVID-19, the purity of the drugs you are buying has:

Increased a lot | Increased somewhat | Increased a little | Stayed the same | Decreased a little | Decreased somewhat | Decreased a lot | N/A

Because of COVID-19, the quality of the drugs you are buying has:

Increased a lot | Increased somewhat | Increased a little | Stayed the same | Decreased a little | Decreased somewhat | Decreased a lot | N/A

Because of COVID-19, the amount you are concerned/worried about Fentanyl contained in the drugs you are buying has:

Increased a lot | Increased somewhat | Increased a little | Stayed the same | Decreased a little | Decreased somewhat | Decreased a lot | N/A

Because of COVID-19, the amount of drugs you use by yourself has:

Increased a lot | Increased somewhat | Increased a little | Stayed the same | Decreased a little | Decreased somewhat | Decreased a lot | N/A

Because of COVID-19, the amount you worry about overdosing has:

Increased a lot | Increased somewhat | Increased a little | Stayed the same | Decreased a little | Decreased somewhat | Decreased a lot | N/A

Because of COVID-19, the number of different triggers for using has (ex. things that can make you want to use):

Increased a lot | Increased somewhat | Increased a little | Stayed the same | Decreased a little | Decreased somewhat | Decreased a lot | N/A

Because of COVID-19, the number of times you have tried not to use but have not been able to stop has:

Increased a lot | Increased somewhat | Increased a little | Stayed the same | Decreased a little | Decreased somewhat | Decreased a lot | N/A
